# Tennis Racket Vibrations and Shock Transmission to the Wrist during Forehand Drive

**DOI:** 10.1371/journal.pone.0132925

**Published:** 2015-07-15

**Authors:** Isabelle Rogowski, Thomas Creveaux, Sylvain Triquigneaux, Pierre Macé, Fabien Gauthier, Violaine Sevrez

**Affiliations:** 1 Université de Lyon, Université Lyon 1, Centre de Recherche et d’Innovation sur le Sport, EA 647, UFRSTAPS, Villeurbanne, France; 2 Babolat VS, Lyon, France; Universidad de Valladolid, SPAIN

## Abstract

This study aimed to investigate the effects of two different racket models and two different forehand drive velocities on the three-dimensional vibration behavior of the racket and shock transmission to the player’s wrist under real playing conditions. Nine tennis players performed a series of crosscourt flat forehand drives at two velocities, using a lightly and a highly vibrant racket. Two accelerometers were fixed on the racket frame and the player’s wrist. The analysis of vibration signals in both time and frequency domains showed no interaction effect of velocity and racket conditions either on the racket vibration behavior or on shock transmission. An increase in playing velocity enlarged the amount of vibrations at the racket and wrist, but weakly altered their frequency content. As compared to a racket perceived as highly vibrating, a racket perceived as lightly vibrating damped longer in the out-of-plane axis of the racket and shorter on the other axis of the racket and on the wrist, and displayed a lower amount of energy in the high frequency of the vibration signal at the racket and wrist. These findings indicated that the playing velocity must be controlled when investigating the vibration loads due to the racket under real playing conditions. Similarly, a reduced perception of vibration by the tennis player would be linked to decreased amplitude of the racket vibration signal, which may concentrate the signal energy in the low frequencies.

## Introduction

Playing tennis exposes the human body to repetitive impacts of the ball with the strings, causing repeated shock waves, moving down the racquet and propagating through the musculoskeletal system. After the ball has left the strings, the player’s arm is beginning to experience not only the forces of the shock wave, but also forces of racquet vibrations [1]. Such vibrations induce discomfort to the player [2]. Although there has been no clinical evidence published to date, repetitive transmission of shocks to the forearm is assumed to affect the upper extremities of the tennis players, and contribute to overuse injury occurrence [3]. In order to limit such risks, equipment companies offer innovative designs, composite materials, and grips or damping devices intended to reduce racket vibrations. While string dampers are claimed to reduce discomfort in the hand and arm, previous studies concluded that they have no other effect on player’s sensations than reducing the sound of impact [2,4]. A multi-layered core damper inserted in the racket handle as for it reduces racket stiffness, and in turn limits the dampening time and amplitudes of vibrations [5].The claimed benefits are mainly based on results obtained by laboratory-testing-based simulation. As a clamped racket may behave differently from a handheld racket [1], the investigation of the racket vibrations and shock transmission under real play condition would be of interest to bring additional knowledge on the player-racket relationship.

Whatever the design and the material components of the racket, two notable areas in the stringbed provide particular benefits to the player [6]. First, the nodal sweet spot is the area of minimal vibration after impact. An impact on the node cancels the fundamental frequency of the racket frame [7]. Second, the center of percussion is the area of minimal shocks to the arm. The tennis players however hit the tennis balls on none of these two optimal areas during real forehand drives. A lack of tennis skills for novice players [8] or a better exploitation of the racket properties for hitting a powerful shot for expert players [9] are the primary reasons for off-centered impacts, which result in increased racket vibrations as well as increased shock transfer to the player’s arm [10]. Most of the vibrations energy detected in the racket subsequently to impact with the ball is related to frequencies below 3000 Hz, with the highest proportion in the out-of-plane direction at frequencies of 80–200 Hz for the racket frame [1]. The majority of frequencies above 500 Hz are attributed to the stringbed [11]. Interestingly, a previous vibration analysis indicated that there is content in the in-plane data frequencies associated with modes of vibration that are predominantly out-of-plane [11]. This suggests that the mode shapes of the racket would be three-dimensional rather than two-dimensional. Moreover, the mechanical coupling of the hand with the racket reduces the frequency of the fundamental mode by 10%, and the vibration dampening time from 175–780 ms to 20–30 ms [12]. The peaks of some frequencies observed during modal analysis are cleared out when playing real forehand drive [11]. Playing conditions such as racket gripping strength, and ball and racket velocities affect the load on the hand [13] and may therefore also influence the racket vibrations and shock transmission. These previous findings highlight the need to evaluate the tridimensional vibration behavior of tennis racket under real playing condition in order to contribute to a better understanding of the shock transmission to the player’s forearm.

The aim of this study was therefore to investigate the effects of two different rackets and forehand drive velocities on the vibration behavior of the racket and shock transmission to the player’s wrist under real forehand drives. It was hypothesized that tennis rackets would be discriminated based on time and frequency descriptors of vibration behavior and that the faster the forehand drive would be, the higher the vibrations of the racket and the shock transmission would be.

## Materials and Methods

Seven male and two female right-handed competitive tennis players (age: 21 ± 2 years, height: 1.76 ± 0.07 m, mass: 68 ± 14 kg, tennis practice: 11 ± 3 years, weekly training: 3.5 ± 1.5 h) volunteered to participate in this study, which was approved by the ethical committee ‘Sud-Est II’. They provided their full written consent to participate. None had a history of injury in the six months preceding the study.

Apart the own racket of each player, two racket prototypes were tested in this study. They were manufactured by Babolat VS (Lyon, France), and were respectively classified as lightly (RL) and highly (RH) vibrating through sensation tests conducted internally. The main characteristics of the racket prototypes were provided by Babolat VS, and are displayed in Table 1.

**Table 1:** Main characteristics of the unstrung and ungripped racket prototypes, with RL for lightly vibrating racket and RH for highly vibrating racket, as defined by sensation tests.

	RL	RH
Mass (g)	263	258
Centre of mass (mm)	352	348
Frame length (mm)	699	685
Swingweight (kg.cm²)	284	279
Polar moment (kg.cm²)	15,3	11,1
Rigidity (N.m)		
Overall	0,10	0.09
Head	0,11	0,11
Handle	0,71	0,73

**Fig 1:**
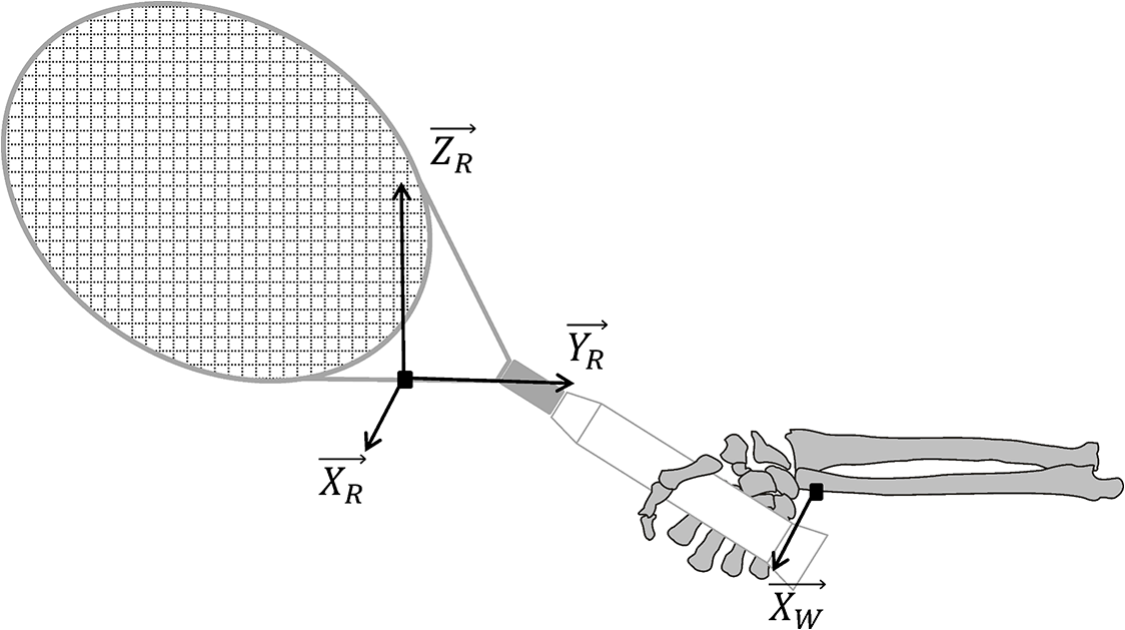
Location and orientation of the accelerometers fixed on the racket throat and the player’s wrist.

All measurements were conducted in an indoor acrylic tennis court, after a 15 min standardized warm-up [14]. One tri-axial and one mono-axis wireless accelerometers (range: ±100 g, sampling rate: 1 kHz, Mega Electronics, Kuopio, Finland) were attached on the throat of the racket and on the ulnar epicondyle of the player’s dominant wrist, respectively. In order to minimize the movement artifacts and improve the mechanical coupling with the structures, the accelerometers were first fixed using double-sided adhesive tape, and then secured with adhesive tape. To allow further interpretation of accelerometer data, the X-axis of the accelerometer located on the racket (X_R_) was set out-of-plane and the Y-axis in-plane (Y_R_). At the wrist, the X-axis (X_w_) of the accelerometer was aligned on X_R_ (Fig 1). The raw data of both accelerometers were recorded using the system WBA (Mega Electronics, Kuopio, Finland).

Players were instructed to first mimic five forehand drives and then hit five series of two sets of 10 forehand drives. The first series was played with their personal rackets at their own playing velocity with their regular grip, and was designed to define the baseline ball post-impact velocity for each player. The other four series resulted from the combination of the two racket prototypes (RL and RH), presented in a random order, with the two velocities, namely baseline velocity reduced by 2.8 m.s^-1^ (V-) and augmented by 2.8 m.s^-1^ (V+). For all series, tennis balls were projected by a ball machine (Airmatic 104, Pop-Lob, Bagneux, France) located behind the baseline of the tennis court. A radar gun (Stalker Pro II, Stalker Radar, Plano, Texas, USA) was placed behind the player to measure ball velocity after impact.

Based on the frequency content of the mimicked forehand drives, the signals from the accelerometers were filtered using a 20Hz high-pass Butterworth filter to remove the motion components [15]. The filtered data of the three-axis of the racket accelerometer and of the wrist accelerometer were filtered and analyzed in both time and frequency domains. For the time domain analysis, the maximal peak-to-peak amplitude, defined as the maximal absolute difference between two consecutive signal peaks, and the damping time, defined as the time during which the signal remained higher than 2 g threshold [15], were computed for all forehand drives. For the frequency domain analysis, a fast Fourier transform algorithm was applied on filtered data. Signal energies were computed as areas under spectrum curves for frequencies up to 200 (E200) and 500 Hz (E500), and the ratio E200/E500 was used as a parameter describing the frequency distribution of the signal energy [15]. The modal frequency was further identified on each of the frequency spectrum. The frequency spectrum components were indented in nine 20Hz bandwidths ranging from 20 to 200Hz, with a step of 20 Hz. For each bin, the energy was expressed as a percentage of the energy contained in the 20–200 Hz range [15].

Nodal impacts, i.e. impacts for which the modal frequency was cleared out, were removed for further analysis. The subsequent statistical analysis was carried out on the non-nodal impacts. All data are presented by mean ± standard error (see S1 File for raw data). The experimental design employed prevented from using a conventional two-way ANalysis Of VAriance (ANOVA) because the assumption of the independence of the observations was not met [16]. Indeed, the dependent variable was measured for a single group of participants across two conditions of ball velocity and of racket. Consequently, a two-way repeated measures ANOVA was performed, in order to test the significativity of the main effects of the two repeated factors (*Racket*: RL vs. RH, and *Velocity*: V- vs V+) and of the interaction between them on the time and frequency descriptors of the accelerometer signal [16]. In case of significant effect of the interaction between playing velocity and racket conditions, post hoc tests were performed with Bonferroni’s correction. All the statistical tests were made using SPSS 11.0. (SPSS, Chicago, IL, USA). The level of significance was set at p≤0.05.

## Results

For all of the studied parameters, ANOVAs revealed no significant effect of the interaction between Velocity and Racket (see S2 File for p values relative to the interaction effect). As a consequence, no *post hoc* pairwise comparisons were needed, and the subsequent results focus only on the *Racket* and *Velocity* main effects considered separately.

The post-impact velocity of the ball was 28.3 ± 1.3 m.s^-1^ with the racket RL and 28.3 ± 1.2 m.s^-1^ with the racket RH for the V- condition, and 33.4 ± 1.4 m.s^-1^ and 33.4 ± 1.3 m.s^-1^, respectively, for the V+ condition. ANOVA revealed no significant effect of the *Racket* and a significant effect of the *Velocity* condition (p<0.001) on post-impact velocity of the ball.

In the time domain, significant effect of *Velocity* (Table 2) was found on the peak-to-peak amplitude for X_R_,Y_R_ and X_w_, as well as on the damping time for all accelerometer axis. Increased post impact velocity of the ball resulted in higher amplitude and longer damping time. Significant effect of *Racket* (Table 3) was found on the peak-to-peak amplitude for the X_R_ axis, and on the damping time for all accelerometer axis. A larger amplitude was observed for RH on the X_R_ compared to RL. A longer damping time was measured for RL on the X_R_, while the damping time was shorter for RL for all other considered axis.

**Table 2:** Mean (± Standard Error) peak-to-peak amplitude, damping time, energy contained in signal up to 500 Hz (E500), up to 200 Hz (E200), E200/E500 ratio, and fundamental frequency for the reduced (V-) and increased (V+) playing velocities as a function of the accelerometer axis.

		Racket X-axis	Racket Y-axis	Racket Z-axis	Wrist X-axis
Peak-to-peak amplitude (g)	V-	163 ± 8 **	45 ± 5 *	85 ± 8	32 ± 4 **
	V+	176 ± 8	52 ± 7	88 ± 9	41 ± 5
Damping time (ms)	V-	85 ± 4 *	56 ± 8 *	51 ± 2 *	33 ± 4 **
	V+	91 ± 5	74 ± 13	56 ± 4	44 ± 6
E500 (AU)	V-	13272 ± 1150 ***	3711 ± 565 **	6629 ± 866 *	2372 ± 280 **
	V+	15520 ± 1373	4555 ± 746	7186 ± 925	3102 ± 470
E200 (AU)	V-	9688 ± 801 **	2557 ± 370 **	4539 ± 694 *	2026 ± 262 **
	V+	11455 ± 1071	3126 ± 490	5010 ± 746	2666 ± 407
E200/E500 (%)	V-	73.6 ± 1.5	70.2 ± 1.4	66.9 ± 2.9	84.5 ± 2.0
	V+	73.8 ± 1.3	70.3 ± 1.1	68.4 ± 2.4	83.5 ± 2.8
Frequency (Hz)	V-	108 ± 3	102 ± 5 **	128 ± 4	65 ± 7
	V+	112 ± 5	86 ± 5	127 ± 4	65 ± 9

* Significant effect of the playing velocity with * for p ≤ 0.05, ** for p ≤ 0.01, and *** for p ≤ 0.001

**Table 3:** Mean (± Standard Error) peak-to-peak amplitude, damping time, energy contained in signal up to 500 Hz (E500), up to 200 Hz (E200), E200/E500 ratio, and fundamental frequency for the lightly (RL) and highly (RH) vibrant rackets as a function of the accelerometer axis.

		Racket X-axis	Racket Y-axis	Racket Z-axis	Wrist X-axis
Peak-to-peak amplitude (g)	RL	161 ± 10 *	45 ± 6	78 ± 9	37 ± 5
	RH	178 ± 9	52 ± 7	95 ± 11	36 ± 4
Damping time (ms)	RL	98 ± 6 ***	61 ± 11 **	43 ± 3 ***	33 ± 5 **
	RH	77 ± 4	70 ± 10	64 ± 4	44 ± 5
E500 (AU)	RL	13464 ± 1423 *	3890 ± 629	6460 ± 981	2871 ± 405
	RH	15328 ± 1176	4376 ± 710	7355 ± 1005	2603 ± 384
E200 (AU)	RL	10313 ± 1162	2795 ± 436	4444 ± 857	2490 ± 363
	RH	10830 ± 743	2888 ± 439	5105 ± 739	2202 ± 337
E200/E500(%)	RL	76.0 ± 0.8 **	73.2 ± 1.6 **	65.3 ± 3.7	83.5 ± 2.8
	RH	71.4 ± 1.8	67.3 ± 1.3	70.0 ± 2.2	84.5 ± 2.0
Frequency (Hz)	RL	92 ± 5 ***	83 ± 8 *	110 ± 4 ***	59 ± 6 **
	RH	128 ± 5	105 ± 5	145 ± 3	72 ± 9

* Significant effect of the racket with * for p ≤ 0.05, ** for p ≤ 0.01, and *** for p ≤ 0.001

In the frequency domain, a significant effect of *Velocity* (Table 2) was found on the energy contained up to 500 Hz and up to 200 Hz, while the ratio between these energies was unchanged for all accelerometer axis. The impacts at V+ generated higher energy for both frequency bands when compared to the impacts at V-. The post-impact velocity of the ball had no effect on the fundamental frequency, except for the Y-axis of the racket accelerometer for which the fundamental frequency was increased for V- in comparison with V+. A significant effect of *Racket* (Table 3) was observed on the energy contained up to 500 Hz for the X_R_ with a lower amount of energy for the RL in comparison with the RH. Significant higher E200/E500 ratio was found for the RL in comparison with RH for the X_R_ and Y_R_. Finally, a significantly lower fundamental frequency was measured for RL in comparison with RH for all accelerometer axis.

**Fig 2:**
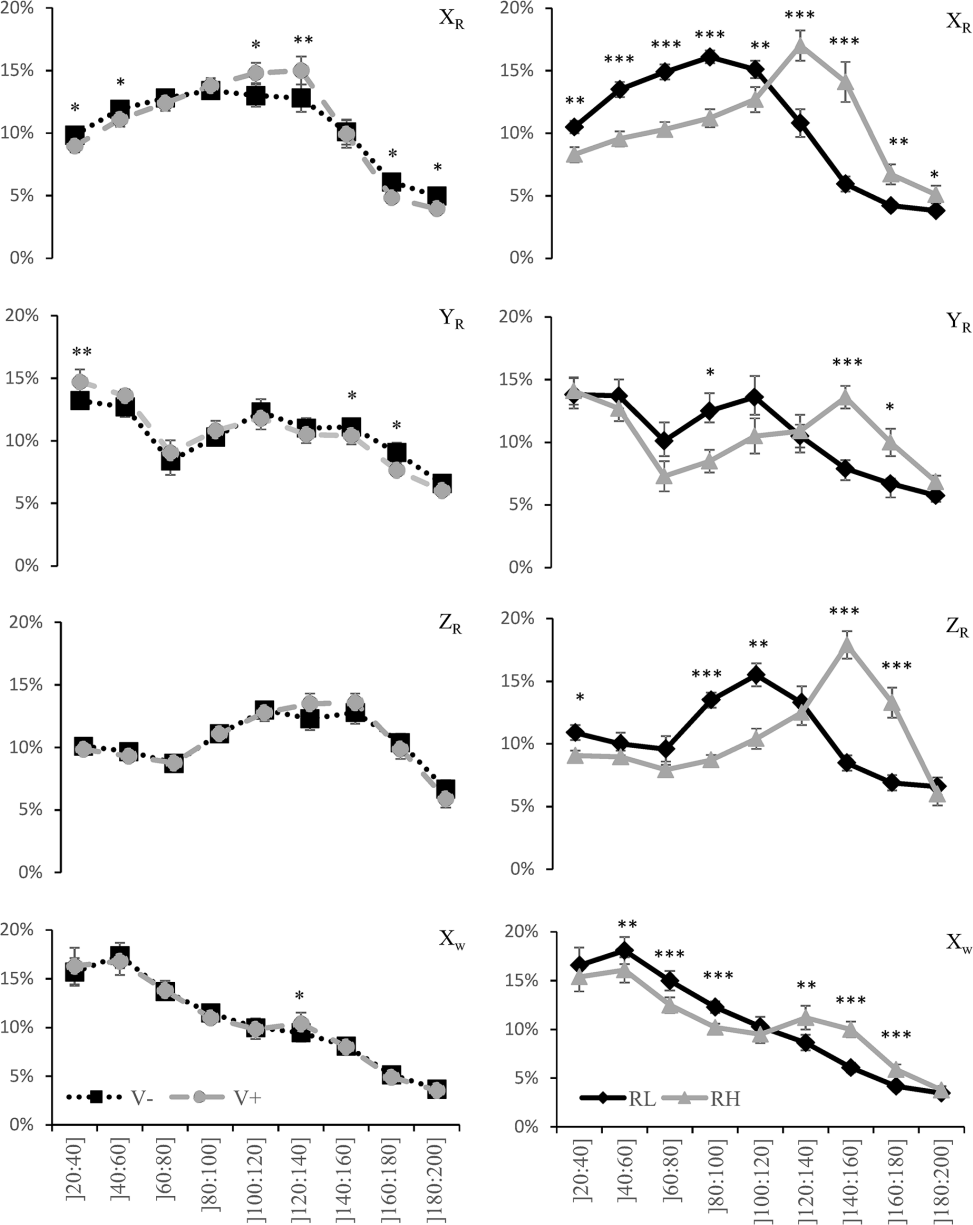
Mean (± Standard Error) relative distribution (%) of the energy contained in the signal up to 200 Hz as a function of the frequency bands on the X-, Y- and Z-axis of the racket accelerometer (XR, YR and ZR, respectively) and X-axis of the wrist accelerometer (XW), for both decreased (V-) and increased (V+) velocity conditions (left), and for both lightly vibrant (RL) and highly vibrant (RH) rackets (right), with * for p≤0.05, ** for p ≤ 0.01, and *** for p ≤ 0.001.

The mean relative distribution of the energy as a function of the frequency bands are presented on Fig 2. A significant effect of *Velocity* was found for the X_R_ and Y_R_ axis with a higher proportion of the energy for low (]20–60 Hz]) and high (]160–200 Hz]) frequency and lower proportion for medium (]100–140]) frequency when playing at V- in comparison with playing at V+. ANOVA revealed a significant effect of *Racket* with a greater proportion of the energy for the low (]20–120 Hz]) frequency and a lower proportion of energy for the high (]120–200 Hz]) frequency on all accelerometer axis when playing with RL in comparison with playing with RH.

## Discussion

This study investigated the effects of post-impact ball velocity and racket characteristics on the three-dimensional vibration behavior of the racket and on the shock transmission to the player’s wrist after real forehand drive impacts. The main results revealed that the playing velocity and racket did not interact to influence either the vibrational behavior of the racket or the shock transmission to the wrist joint. An increased playing velocity increased the amount of vibrations at the racket and wrist joint, but weakly altered the frequency content of the vibration signal. A racket perceived as lightly vibrant damped vibration in a longer time on the out-of-plane axis of the racket and in a shorter time on the other axis of the racket and wrist as compared to a racket perceived as highly vibrant. It also displayed a lower amount of energy in the high frequency of the vibration signal at the racket and the wrist.

A part of the large amount of energy generated by the impact of the ball in the stringbed transforms into energy of vibration, which is transferred to the player’s hand and arm [8]. The primary source of player’s discomfort comes from the frame vibration [2]. The larger amount of vibration signal was observed in the out-of-plane direction of the racket (X_R_ in our study), while non-negligible signal was reported for the two other directions of the racket, hence confirming that the mode shapes of the racket were three-dimensional rather than two-dimensional [11]. In addition, the characterization of the vibration behavior of the racket frame is mainly based on the damping time [5] and the fundamental frequency [12], while that of the shock transmission to the player’s wrist is on peak-to-peak acceleration and integrated acceleration [10]. Our values for the fundamental frequency observed on the out-of-plane of the racket (Tables 2 and 3) were in the previously reported range 80–200 Hz [1]. Similarly, our values for the integrated acceleration at the wrist joint (Tables 2 and 3) were close to the 2200 AU measured by Hennig et al. [10]; these previous values being obtained from impact on handheld rackets without arm motion. However, we found higher values for the racket damping time (Tables 2 and 3) and peak-to-peak acceleration at the wrist joint (Tables 2 and 3) as compared to the previous 0.30–0.85 ms [5–6] and 20 g [10], respectively measured from impact in clamped and handheld racket conditions. Such differences may be explained by the mechanical coupling of the hand with the racket, as well as by the racket swing and rotation, all of which affecting the physical responses of the racket frame [9]. This result suggests that the vibration behavior of the racket and the shock transmission to the hand and arm should be investigated under real playing condition in order to contribute to the understanding of the vibration loads sustained by tennis players through real ball impact.

The primary result of the current study indicated that the playing velocity and the racket type may not interact to affect the vibration behavior of the racket and the shock transfer to the wrist joint. This suggests a cumulative effect of playing and racket conditions on the vibration stress experienced by the tennis player. An increased playing velocity resulted in increased signal amplitude, increased damping time and increased signal energy (Table 2), with minimal changes in the distribution of the signal frequency content (Fig 2). As playing at high velocity is facilitated by off-center impact [9], i.e. impacting the ball at location on the stringbed other than the nodal sweet spot or the center of percussion, it may generate increased vibration amplitude of the racket frame and in turn enlarge the forced oscillations at the wrist joint [10] whatever the racket being used. The choice of the racket may thus appear to be important to limit the amount of vibration.

The rackets used to perform forehand drives in this study differed by less than 3% for the mass, center of mass, frame length, swingweight, as well as head and handle rigidity (Table 1). The racket perceived as lightly vibrant however presented higher polar moment (+36%) and overall rigidity (+11%) than the racket perceived as highly vibrant (Table 1). The racket perceived as lightly vibrant was characterized by reduced peak-to-peak amplitude and increased damping time in the out-of-plane direction (Table 3), hence demanding less time to damp the vibrations at the wrist joint as compared to a racket perceived as highly vibrant. These findings may confirm that a stiff racket may absorb less energy, thus likely resulting in lower racket vibration amplitudes and less vibration transfer to the human body [1]. The lightly vibrant racket was also characterized by a larger amount of signal at low frequencies (Table 3)- i.e. larger proportion of signal energy attributed to the first than to the second vibration mode of the racket—and by a reduced fundamental frequency, affecting the frequency mode at the wrist joint (Table 3). When focusing on the frequency bands primarily felt by a player (Fig 2), our results clearly showed that, in all the three directions, a lightly vibrant racket concentrated more energy in the low frequencies (20 to 120 Hz), while a highly vibrant racket concentrated more energy in the 120–200 Hz frequencies. Similar differences in the signal energy distribution were observed at the wrist joint (Fig 2). A reduced perception of the racket vibration by the tennis player might be related with the attenuation capacity of the human soft-tissue. It would seem that the attenuation of the vibration might be linked to the viscoelastic properties of the soft-tissues [17]. The dynamic viscosity would be the most efficient mechanism to attenuate vibration, but would mainly act on the low frequencies of the vibration signal [17]. The increased frequency of vibrations may decrease the soft-tissue dynamic viscosity, hence limiting the absorption capacities of the soft-tissues in response to off-centered impact during forehand drive. Although the effects of such impacts on the anatomical structures remain unclear, it could be hypothesized that repeated high magnitude shocks may place large stress on the anatomical structures of the tennis players, resulting in an increased risk of injury and degenerative disease.

This study presents some limitations that warrant discussion. Aside from the traditional issues related to the coupling of sensor and racket/skin, the device used to measure the vibration behavior and the shock transfer is primary recommended for human motion analysis, hence explaining the low frequency for the data collection (1000 Hz). The lack of precise signal synchronization between the accelerometers on the racket and wrist joint did not allow an exploitation of the time-frequency domain parameters. Nevertheless, this tool may be considered appropriate as player’s perception is mainly focused on the low-frequency fundamental frame vibration (80–200 Hz). A second limitation is the lack of measurement of both handgrip force and ball impact location in the stringbed. Such parameters are known to affect the vibration behavior of the frame and the shock transmission; it was then assumed that the players involved in the current study were skilled enough to reproduce handgrip force and ball impact location. Each player was therefore used as his own reference for the paired comparisons, hence limiting the methodological bias in regards to the players' intra-individual variability. A third limitation concerns the choice of the rackets. As no information were available in the literature regarding *in situ* discrimination of racket behavior, we intentionally tested two rackets having extreme vibration behaviors. Further studies evaluating rackets with unknown vibration behavior are then encouraged. Finally, the main goal of this study was to screen for potential parameters allowing the racket vibration behavior to be discriminated. The large number of vibration parameters studied led to many simultaneous statistical tests thus possibly increasing the number of false rejections. However, this study was the first to involve the dynamics of the human player in the analysis while simultaneously recording both the racket 3D-vibrational behavior and the shock transmission to the wrist joint. The results indicated that the playing velocity must be controlled when investigating the vibration loads due to the racket under real playing condition. The findings also revealed that the choice of the racket may be of primary importance to reduce the amount of racket vibrations as well as to limit the shock transfer to the player’s arm.

In conclusion, the results of this study showed no interrelated effect of playing velocity and racket tennis condition on the racket vibration behavior and shock transmission. An increase in playing velocity enlarged the amount of vibrations at the racket and wrist joint, but weakly altered the frequency content of the vibration signal. A racket perceived as lightly vibrant damped longer on the out-of-plane axis of the racket and shorter on the other axis of the racket and wrist, and displayed lower amount of energy in the high frequency of the vibration signal at the racket and wrist, than a racket perceived as highly vibrant. Further studies are needed to investigate the influence of the spin of shot, such as topspin, and the racket properties, such as mass or stiffness, on racket vibration behavior and shock transmission to the arm in order to provide new knowledge on the vibration stress sustained by tennis players during real play.

## Supporting Information

S1 File Raw data. (XLS)

S2 File p values relative to the interaction effect obtained with the ANOVAs. (XLSX)

## References

[1] Hennig EM. Influence of racket properties on injuries and performance in tennis. Exerc Sport Sci Rev. 2007;35: 62–66. 17417052 10.1249/JES.0b013e31803ec43e

[2] Stroede CL, Noble L, Walker HS. The effect of tennis racket string vibration dampers on racket handle vibrations and discomfort following impacts. J Sports Sci. 1999;17: 379–385. 10413265 10.1080/026404199365894

[3] Li F-X, Fewtrell D, Jenkins M. String vibration dampers do not reduce racket frame vibration transfer to the forearm. J Sports Sci. 2004;22: 1041–1052. 15801498 10.1080/02640410410001729982

[4] Timme N, Morrison A. The mode shapes of a tennis racket and the effects of vibration dampers on those mode shapes. J Acoust Soc Am. 2009;125: 3650–3656. 10.1121/1.3126343 19507947

[5] Ferrara L, Cohen A. A Mechanical Study on Tennis Racquets to Investigate Design Factors that Contribute to Reduced Stress and Improved Vibrational Dampening. Proc Eng. 2013;60: 397–402.

[6] Brody H. Models of tennis racket impacts. Int J Sport Biomech. 1987;3: 293–296.

[7] Cross R. The sweet spots of a tennis racquet. Sports Eng. 1998;1: 63–78.

[8] Wei SH, Chiang JY, Shiang TY, Chang HY. Comparison of shock transmission and forearm electromyography between experienced and recreational tennis players during backhand strokes. Clin J Sport Med. 2006;16: 129–135. 16603882 10.1097/00042752-200603000-00008

[9] Nass D, Hennig EM, Schnabel G. Ball impact location on a tennis racket head and its influence on ball speed, arm shock and vibration. In: Proceedings II of the XVI ISBS Symposium; 1998 Jul 21–25: Konstanz (Germany). UVK-Universitätsverlag GmbH; 1998. p. 229–232.

[10] Hennig EM, Rosenbaum D, Milani TL. Transfer of tennis racket vibrations onto the human forearm. Med Sci Sports Exerc. 1992;24: 1134–1140. 1435161

[11] Banwell G, Roberts JR, Mohr S, Rothberg SJ. Identifying the Modes Excited in a Tennis Racket by a Forehand Drive. Topics in Modal Analysis II. 2012;6: 649–655.

[12] Brody H. Vibration damping of tennis rackets. Int J Sport Biomech. 1989;5: 451–456.

[13] Knudson D. Factors affecting force loading on the hand in the tennis forehand. J Sports Med Phys Fitness. 1991;31: 527–531. 1806729

[14] Genevois C, Frican B, Creveaux T, Hautier C, Rogowski I. Effects of two training protocols on the forehand drive performance in tennis. J Strength Cond Res. 2013;27: 677–682. 10.1519/JSC.0b013e31825c3290 22592176

[15] Creveaux T, Sevrez V, Coste B, Rogowski I. Methodological contribution to study the vibratory behaviour of tennis rackets following real forehand drive impact. Comp Meth Biomech Biomed Eng. 2014;17: S150–151. 10.1080/10255842.2014.931610 25074209

[16] Howell DC. Repeated-measures designs In Howell DC, editor. Statistical method for psychology. Wadsworth: Cengage Learning: 2013 pp. 457–506.

[17] Sarvazyan A, Rudenko O, Aglyamov S, Emelianov S. Muscle as a molecular machine for protecting joints and bones by absorbing mechanical impacts. Med Hypotheses. 2014;83: 6–10. 10.1016/j.mehy.2014.04.020 24810676 PMC4112738

